# The Prevalence and Impact of Hepatic Steatosis on Response to Direct-Acting Antiviral Therapy in HIV–HCV Coinfection

**DOI:** 10.3390/biology9040087

**Published:** 2020-04-24

**Authors:** Leigh P. Johnson, Richard K. Sterling

**Affiliations:** 1 Department of Internal Medicine, Virginia Commonwealth University, Richmond, VA 23298-0341, USA; Leigh.Johnson@vcuhealth.org; 2 Division of Gastroenterology, Hepatology and Nutrition, Virginia Commonwealth University, Richmond, VA 23298-0341, USA; 3 Division of Infectious Diseases, Virginia Commonwealth University, Richmond, VA 23298-0341, USA

**Keywords:** hepatic steatosis, sustained virologic response (SVR), direct-acting antiviral (DAA) therapy, hepatitis C virus (HCV), human immunodeficiency virus (HIV)

## Abstract

(1) Background: Direct-acting antiviral therapy for chronic hepatitis C virus (HCV) infection is associated with high sustained virologic response (SVR) and overcomes negative predictive factors, including steatosis, in patients without human immunodeficiency virus (HIV) coinfection. The impact of steatosis on SVR in patients with HIV–HCV coinfection is unknown. (2) Methods: A retrospective analysis of patients treated with direct-acting antivirals was performed. Demographic, laboratory and direct-acting antiviral regimen data were prospectively collected. Metabolic syndrome and its components—diabetes mellitus, hypertension, dyslipidemia and obesity—were assessed. Hepatic steatosis (≥5%) was defined by liver biopsy or controlled attenuation parameter (CAP) measurement during vibration-controlled transient elastography (VCTE). (3) Results: A total of 151 HIV–HCV-coinfected patients on combined antiretroviral therapy and direct-acting antiviral therapy were included in this analysis. Prevalence of steatosis by liver biopsy (n = 34) or CAP (≥263 db/m) during VCTE (n = 92) was 27% and was independently associated with obesity (OR 3.11; 95% CI 1.43–6.82; *p* = 0.004) and the metabolic syndrome (OR 1.08; 95% CI 1.01–0.15; *p* = 0.01). The overall SVR rate (n = 148) was 95% and was not impacted by the presence of steatosis (*p* = 0.42). (4) Conclusions: Hepatic steatosis is common in HIV–HCV coinfection, correlates with obesity and the metabolic syndrome and does not impact SVR.

## 1. Introduction

Hepatic steatosis is common in patients living with human immunodeficiency virus (HIV), including those coinfected with chronic hepatitis C virus (HCV) [1,2]. In studies of HIV patients at risk for steatosis (e.g., increased liver enzymes) without HCV using liver histology as reference standard, steatosis was detected in 60%–69% [2,3,4,5] compared to 23%–75% of those with HIV–HCV coinfection [1] and 40%–86% of those with chronic HCV without HIV [6]. Similar to those without HIV, the development of steatosis in patients with HIV is often secondary to the presence of the metabolic syndrome and its components (diabetes, obesity, hypertension, and dyslipidemia), HIV-related lipodystrophy, microbial dysbiosis and mitochondrial damage leading to insulin resistance and dyslipidemia [7,8,9]. Furthermore, some of the HIV therapies, specifically older nucleoside/nucleotide analogue reverse transcriptase inhibitors and protease inhibitors, cause lipolysis, and this increase in free fatty acids results in mitochondrial dysfunction [10]. These medications also lead to insulin resistance via modulation of adipose and intestinal tissue; leptin, interleukin-6, interleukin-1, tumor necrosis factor alpha and peroxisome proliferator-activated receptor gamma are increased where adiponectin is decreased [7,11]. The mechanism of hepatic steatosis in patients with HCV genotype 1 (GT1) as well as HIV is believed to be through insulin resistance [12]; hence, the observed association of steatosis with obesity in this cohort. A meta-analysis of 34 studies found a positive correlation between HCV and the development of diabetes [13]. It is plausible that via increased insulin resistance, the presence of diabetes is associated with steatosis; interestingly, this association was not found in this cohort. Furthermore, there is evidence that HCV itself, especially genotype 3 (GT3), can lead to steatosis [14]. In addition to factors mentioned above, high alcohol consumption and HCV GT3 are risk factors associated with steatosis in HCV patients [6,15,16,17,18,19].

Liver biopsy is the reference standard for diagnosing hepatic fibrosis and steatosis; however, it is invasive, expensive and affected by biopsy size (sampling error) and interobserver variation among pathologists [20]. Consequently, non-invasive serum-based tests and imaging techniques, such as vibration-controlled transient elastography (VCTE) to measure fibrosis with controlled attenuation parameter (CAP) measurement to assess hepatic steatosis, respectively, have replaced liver biopsy in most centers [21,22]. However, because VCTE with CAP may not be available in many clinical settings, other non-invasive serum-based assessments of steatosis, such as the hepatic steatosis index (HSI) have been developed [23,24] but not assessed in those with HIV.

The presence of steatosis resulted in lower sustained virologic response (SVR) during the interferon-alpha treatment era of HCV infection [25,26]. The development of direct-acting antiviral (DAA) medications revolutionized the treatment of HCV infection, including those with HIV, resulting in high SVR (90%–95%) [27,28]. Past negative predictive factors associated with non-response when treated with interferon therapies, such as the metabolic syndrome, insulin resistance, advanced fibrosis, black race, GT1, and HIV–HCV coinfection, do not appear to be risk factors for relapse in the DAA era [27,28,29]. Because hepatic steatosis does not appear to be a risk factor for DAA treatment failure in those with HCV monoinfection [30], it is hypothesized that steatosis will not impact SVR in HIV–HCV coinfection.

The impact of hepatic steatosis on SVR in HIV–HCV-coinfected patients receiving DAA therapy is unknown. To address this gap in knowledge, the primary aims of the current study were to determine the prevalence and risk factors of hepatic steatosis in a cohort of HIV–HCV-coinfected patients undergoing DAA therapy and the impact of steatosis on SVR. The secondary aim was to assess the sensitivity and specificity of the HSI versus CAP for detecting steatosis to determine its utility in settings where VCTE and CAP are not available.

## 2. Materials and Methods

### 2.1. Study Design and Participants

This was a retrospective analysis of adult patients treated at Virginia Commonwealth University Health with DAAs between December 2014 and December 2018. Participants provided written informed consent prior to enrollment. All patients were treated with DAAs in accordance with American Association for the Study of Liver Disease/Infectious Disease Society of America (AASLD-IDSA) Guidelines [31]. The study was approved by the HCV Treatment Registry institutional review board (IRB). Adult patients with HIV–HCV coinfection adherent with a DAA regimen were eligible for inclusion. Exclusion criteria included active evaluation for organ transplant, significant hepatic disease (e.g., acute hepatitis, decompensated cirrhosis), uncontrolled HIV (CD4 count <200 cells/mm^3^), and active alcohol use (men who reported drinking >4 drinks on any day or >14 drinks per week; and women who reported drinking >3 drinks on any day or >7 drinks per week; a standard drink was defined as 14 grams of ethanol) [32].

### 2.2. Study Outcomes

The primary outcomes were to determine the prevalence and risk factors of hepatic steatosis in a cohort of HIV–HCV-coinfected patients undergoing DAA therapy and the impact of steatosis on SVR 12 weeks after completion of therapy (SVR-12). The secondary aim was to assess the sensitivity and specificity of the HSI versus CAP for detecting steatosis to determine its utility in settings where VCTE and CAP are not available.

### 2.3. Data Collection and Laboratory Analysis

To ascertain the primary outcome, the presence of hepatic steatosis was measured by liver biopsy (≥5%) (n = 34), CAP (≥263 db/m) [33] during VCTE (n = 92) and the serologic-based testing score, the hepatic steatosis index (HSI) (>41) (n = 151) (HSI = 8 × (alanine aminotransferase level (U/L)/aspartate aminotransferase level (U/L) ratio) + BMI (+2, if female; +2, if diabetes mellitus)) [23]. The CAP threshold of 263 dB/m was validated in patients with non-alcoholic fatty liver disease (NAFLD) and chosen for this study because of its high sensitivity (90%) for detecting at least five percent steatosis compared to liver histology [34]. Lower CAP thresholds (238 dB/m [35] and 248 dB/m [33]) have also been validated for evaluating the presence of hepatic steatosis in patients with NAFLD [33,35] and HCV [33]. It is important to recognize that none of these thresholds, including 263 dB/m used in this study, are validated in patients with HIV or HIV–HCV coinfection. The lower CAP thresholds were included in a sensitivity analysis with a HSI > 41. Liver fibrosis was assessed by liver biopsy (n = 34) and VCTE (n = 117). Significant liver fibrosis by VCTE was defined as >9.0 kPa [20,36,37]. A higher threshold of 11 kPa was also used to define advanced fibrosis [34,38,39,40]. Additionally, the aspartate aminotransferase to platelet ratio index (APRI = (aspartate aminotransferase level (U/L)/aspartate aminotransferase upper limit of normal (U/L))/(platelet count (×10^9^/L) × 100) and fibrosis 4 index for liver fibrosis (FIB-4 = (age (years) × aspartate aminotransferase level (U/L))/(platelet count (×10^9^/L) × square root of alanine aminotransferase level (U/L)) were calculated based on baseline data [41]. Demographic and baseline characteristics including, age, gender, race, HCV genotype, liver fibrosis grade by biopsy and/or VCTE, liver CAP score, body mass index (BMI), history of obesity, diabetes, hypertension, the metabolic syndrome, hepatitis B virus (HBV), and alcohol use as well as laboratory, HIV combination antiretroviral therapy (cART) and DAA regimen data were prospectively collected. Diabetes and hypertension were classified as present if patients were taking antidiabetic or antihypertensive medications. Obesity was defined as BMI ≥ 30 kg/m^2^. The metabolic syndrome was defined as the presence of at least three of the following: BMI ≥ 30 kg/m^2^, diabetes, hypertension, or dyslipidemia (triglyceride level ≥ 150 mg/dL) [42]. SVR was defined as undetectable HCV RNA 12 weeks after completing DAA treatment (SVR-12).

### 2.4. Statistical Analyses

Descriptive statistics are presented using means and standard deviation (SD) for normally distributed continuous variables or medians and interquartile range (IQR) when data were skewed. Categorical variables were expressed as percent. For the primary analysis, the percent of participants who achieved SVR-12 was calculated. Univariate analysis was done to compare SVR-12 in those with and without hepatic steatosis. Differences in continuous variables were assessed by the *t*-test or the Mann–Whitney U test while differences in categorical variables were assessed by chi-square or Fisher exact test as appropriate. Factors on univariate analysis with *p* < 0.2 were used in the multivariate logistic regression analysis to identify independent factors associated with SVR-12 (the primary outcome) and hepatic steatosis (by biopsy or CAP > 263 dB/m). The relationship between the HSI and CAP was assessed by Spearman’s correlation. A sensitivity analysis of detecting ≥5% steatosis by a HSI > 41 versus various CAP thresholds (238, 248, and 263 dB/m) was used to determine the sensitivity, specificity, positive predictive value (PPV) and negative predictive value (NPV) of the HSI. All statistical analyses were conducted with JMP14 Pro. *p* < 0.05 (2-sided) was considered statistically significant.

## 3. Results

### 3.1. Study Participants

One hundred and fifty one HIV–HCV-coinfected patients on cART and DAAs were included in this analysis (Table 1). The median age was 57 years, 76% were male, 84% were black, 97% were HCV GT1, and 5% were infected with hepatitis B virus. The median alanine transaminase (ALT) level was 54 units per liter (U/L), the median body mass index (BMI) was 27 kg per square meter (kg/m^2^), and the median cluster of differentiation 4 (CD4) count was 559 (IQR 390–830) cells per cubic millimeter (cells/mm^3^). The prevalence of diabetes mellitus, hypertension, and obesity was 21%, 39%, and 34%, respectively, while metabolic syndrome was present in 33% of participants. The predominant cART used included a backbone nucleoside reverse transcriptase inhibitor (NRTI) (91%) in combination with a non-nucleoside reverse transcriptase inhibitor (NNRTI) (20%), protease inhibitor (PI) (24%), or an integrase inhibitor (II) (69%). The majority of those on a NRTI were on tenofovir (68%) or abacavir (25%) combined with emtricibine or lamivudine, while the majority of those on a PI were on darunavir (39%) or atazanavir (25%) combined with ritonavir or cobisistat. The predominant DAA used was ledipasvir/sofosbuvir (79%) with ribavirin (22%) for 12 weeks; the remaining participants received sofosbuvir/velpatasvir (8%), elbasvir/grazoprevir (6%), or glecaprevir/pibrentasvir (2%) for 12 weeks.

### 3.2. Primary Outcomes

The liver disease characteristics are shown in Table 2. In participants who underwent liver biopsy (n = 34), 26% had mild fibrosis (score of 1), 30% had moderate or severe fibrosis (score of 2 or 3), and 43% had cirrhosis (score of 4). The prevalence of significant (>9 kPa) and advanced hepatic fibrosis (>11 kPa) by VCTE was 48% and 40%, respectively, with a median kPa of 8.65 overall. The prevalence of hepatic steatosis (≥5%) by liver biopsy (n = 34) or CAP (≥263 db/m) during VCTE (n = 92) was 27%. Using different CAP thresholds, the prevalence of steatosis increased as the CAP threshold decreased (15% with CAP ≥ 263 dB/m to 35% with CAP ≥ 238 dB/m).

Table 3 compares those with and without steatosis. On univariate analysis, steatosis was associated with increasing BMI (*p* = 0.013), obesity (*p* = 0.004), dyslipidemia (triglyceride level ≥ 150 mg/dL) (*p* = 0.01), and the metabolic syndrome (*p* = 0.01). On multivariate analysis, steatosis was independently associated with obesity (OR 3.11; 95% CI 1.43–6.82; *p* = 0.004) and the metabolic syndrome (OR 1.08; 95% CI 1.01–0.15; *p* = 0.01) but not with age, diabetes mellitus, dyslipidemia, hypertension, ALT, or cART type.

**Figure 1:**
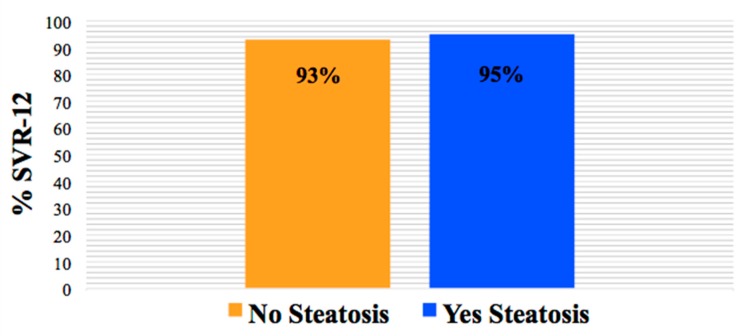
SVR-12 rate by the presence and absence of hepatic steatosis (*p* > 0.05).

SVR-12 data were available for 98% of the participants (n = 148). The overall SVR-12 rate in those with hepatic steatosis was 95% and similar to those without steatosis (93%). In addition, SVR-12 was not impacted by patient demographics, cART regimen, or liver fibrosis (all *p* > 0.05) (Figure 1).

### 3.3. Secondary Outcomes

**Figure 2:**
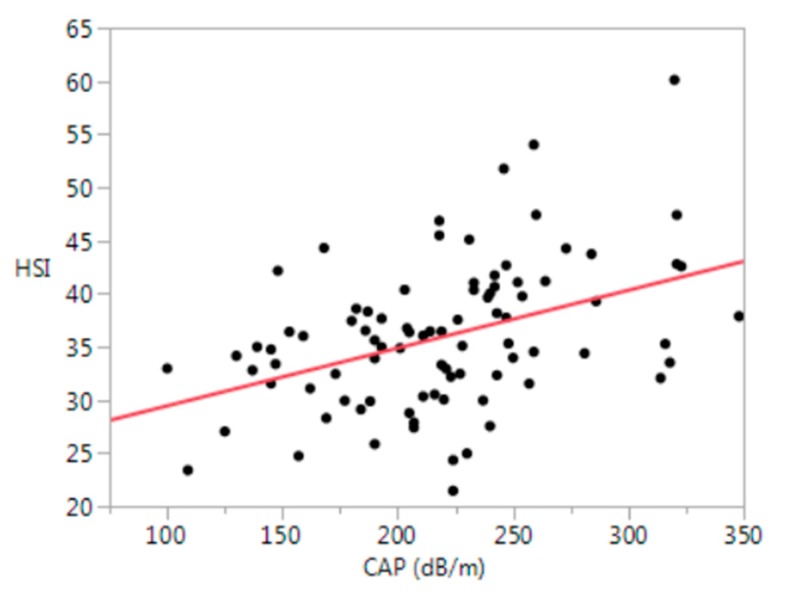
Relationship between the HSI and CAP (r = 0.41, *p* < 0.0001). HSI = hepatic steatosis index (8 × (alanine aminotransferase level (U/L)/aspartate aminotransferase level (U/L)ratio) + BMI (+2, if female; +2, if diabetes mellitus)); CAP = controlled attenuation parameter; dB/m = decibel per meter.

Hepatic steatosis by the HSI (>41) was present in 24% of participants and strongly correlated with CAP (≥263 db/m) (r = 0.41, *p* < 0.0001) (Figure 2).

However, the HSI (>41) had low sensitivity (39%) and positive predictive value (PPV) (50%) with good specificity (90%) and negative predictive value (NPV) (86%) for detecting steatosis versus CAP (≥263 db/m). Table 4 shows the sensitivity analysis of the HSI (>41) when CAP thresholds are lower. As the CAP threshold is lowered, the sensitivity and NPV increases and the specificity and PPV decreases. The HSI has the highest sensitivity and NPV at a CAP of 238 dB/m, and the highest specificity and PPV at a CAP of 263 dB/m.

## 4. Discussion

The prevalence of hepatic steatosis is common in those with HIV, including those with HIV–HCV coinfection [1,2,3,4,5], and may be due to the high prevalence of the metabolic syndrome in those with HIV [43]. The prevalence of steatosis in the present cohort (27%) is at the lower end of the spectrum compared to past reports on HIV–HCV coinfection (23–75%) [1] and is related to BMI, obesity, and dyslipidemia, but not to diabetes and hypertension—two components of the metabolic syndrome. The prevalence of steatosis may be lower in this cohort because of the high proportion of black participants (81%) who have been observed to have a lower rate of steatosis compared to that in white and Hispanic individuals [44]. This discrepancy may also be due to the small sample size of the cohort. Steatosis was defined by CAP in the majority of patients, and the cutoff of 263 dB/m was chosen for maximum sensitivity to minimize the chance of falsely identifying those without steatosis [34]. When the threshold was reduced to 238 dB/m, the prevalence increased to 35%.

In the interferon era, hepatic steatosis negatively impacted SVR [25,26]. The development of DAAs has dramatically increased the ability to achieve SVR in chronic HCV [27,28,29,30]. Although DAAs have negated the presence of steatosis on SVR in those with HCV monoinfection [30], less is known about those with HIV–HCV coinfection. In this cohort, the SVR-12 rate was high, similar to that found in studies on participants with HCV monoinfection (90%–95%) [27,29], and not impacted by the presence of hepatic steatosis.

Liver biopsy, the gold standard for assessment of fibrosis and steatosis, has now been replaced by non-invasive methods such as the fibrosis 4 (FIB-4) index for liver fibrosis, the HSI, and VCTE [20,21,22,41]. In addition to biopsy and VCTE, the HSI was used to detect steatosis in this cohort because all of its components were collected as part of routine care. Although the HSI (>41) correlated with CAP (≥263 dB/m) as a continuous variable and had reasonable specificity compared to CAP (90% at a CAP of 263 dB/m), it had poor sensitivity and moderate positive predictive value, suggesting that it may be most useful to rule in steatosis rather than rule it out. The negative predictive value of the HSI (>41) for detecting steatosis was highest at a low CAP threshold (≥238 db/m). Based on the results, the HSI should not replace VCTE with CAP and should be used with caution in HIV–HCV-coinfected patients [23,24].

There are numerous limitations to this retrospective analysis. The cohort was predominantly black, male, and GT1—reflective of our institution’s coinfected population [18]; however, the results may not be generalizable to all those with HIV–HCV coinfection. Because SVR was high and few participants were infected with GT3, the interactions among GT3, steatosis, and SVR were not assessed. Past alcohol use among participants, prior to enrollment, was not recorded. In addition, liver biopsy was not performed on all participants; thus, VCTE with CAP was used to detect steatosis in the majority of patients. Because CAP was not available until 2016, CAP was not performed during VCTE on all patients undergoing DAA therapy. Although the HSI was used as another non-invasive measure of steatosis, it was not validated for patients with HIV and needs to be used with caution. The HSI was compared with CAP, but not with histology. The presence of non-alcoholic steatohepatitis (NASH) was not evaluated in this cohort. Lastly, given the high SVR-12 rate, a much larger sample size would be needed to detect significant differences in SVR-12 between those with and without steatosis.

## 5. Conclusions

Although hepatic steatosis is common in those with HIV–HCV coinfection undergoing DAA therapy and related to obesity, it has no impact on SVR. The HSI should not replace VCTE with CAP for detecting the presence of steatosis in HIV–HCV-coinfected patients. More research is needed to understand how eradication of HCV with DAAs affects hepatic steatosis and the potential progression to fibrosis, cirrhosis, and hepatocellular carcinoma [27]. Long-term follow-up of this HIV positive SVR-12 cohort to determine the impact of underlying steatosis on liver- and non-liver-related morbidity and mortality is ongoing.

## Figures and Tables

**Table 1:** Demographic and laboratory characteristics of the cohort.

Variable (n = 151)	Mean/%	Standard Deviation	Median	Interquartile Range
Age (years) ^	54.9	9.5	57	50–61
Gender (% male)	76			
Race (% black)	84			
HCV genotype (% type 1)	97			
ALT (U/L) ^	69.8	68.8	54	37–77
Platelet count (×10^9^/L) ^	172	67.8		
HBV (%)	5			
BMI (kg/m^2^) ^	27.7	6.14	26.7	23.8–30.7
Obesity (% BMI ≥ 30 kg/m^2^)	34			
Diabetes (%)	21			
Hypertension (%)	39			
Metabolic syndrome (%) *	32.4			
CD4 count (cells/mm^3^) ^	632	332	559	390–830
cART				
NRTI (%)	91			
NNRTI (%)	20			
PI (%)	24			
II (%)	69			

^ Mean +/− standard deviation. * The presence of at least three of the following: BMI ≥ 30 kg/m^2^, diabetes, hypertension, or dyslipidemia (triglyceride level ≥ 150 mg/dL). HCV = hepatitis C virus; ALT = alanine aminotransferase; HBV = hepatitis B virus; VCTE = vibration-controlled transient elastography; kPa = kilopascal; BMI = body mass index; CD4 = cluster differentiation 4; cART = combined antiretroviral therapy; NRTI = nucleoside reverse transcriptase inhibitor; NNRTI = non-nucleoside reverse transcriptase inhibitor; PI = protease inhibitor; II = integrase inhibitor.

**Table 2:** Liver disease characteristics of the cohort.

Variable (n = 151)	Mean/%	Standard Deviation	Median	Interquartile Range
Steatosis (%) *	27			
HSI ^	36.6	7.48	36.05	31.54–40.71
HSI > 41 (%)	24			
CAP (dB/m) (n = 92) ^	217.5	51.7	219	186–246.75
CAP > 263 (dB/m) (%)	15			
CAP > 248 (dB/m) (%)	25			
CAP > 238 (dB/m) (%)	35			
Fibrosis (% 0/1/2/3/4) **	1/26/12/18/43			
APRI ^	1.002	0.99	0.77	0.4–1.21
FIB-4 ^	3.48	2.77	2.57	1.69–4.16
VCTE (kPa) (n = 118) ^	12.4	10.15	8.65	5.9–14.3
VCTE > 9 (kPa) (%)	48			
VCTE > 11 (kPa) (%)	40			

^ Mean +/− standard deviation. HSI = hepatic steatosis index (8 × (alanine aminotransferase level (U/L)/aspartate aminotransferase level (U/L)ratio) + BMI (+2, if female; +2, if diabetes mellitus)); CAP = controlled attenuation parameter; dB/m = decibel per meter; APRI = aspartate aminotransferase to platelet ratio index ((aspartate aminotransferase level (U/L)/aspartate aminotransferase upper limit of normal (U/L))/(platelet count (×10^9^/L) × 100); FIB-4 = fibrosis 4 index for liver fibrosis ((age (years) × aspartate aminotransferase level (U/L))/(platelet count (×10^9^/L) × square root of alanine aminotransferase level (U/L)); VCTE = vibration-controlled transient elastography; kPa = kilopascal. * By liver biopsy or CAP > 263 (dB/m). ** By liver biopsy: 0 = no fibrosis; 1 = mild fibrosis; 2 = moderate fibrosis; 3 = severe fibrosis; 4 = cirrhosis.

**Table 3:** Demographic, laboratory and liver disease characteristics of the cohort by the presence or absence of steatosis.

Variable (n = 142)	No steatosis (n = 104)	Steatosis (n = 38)	*p* Value	MLR *p* Value/OR/CI
Age (years) ^	55 +/− 10	57 +/− 10	0.17	
Gender (% male)	77	74	0.64	
Race (% black)	81	85	0.56	
Fibrosis (% 0/1/2/3/4) *	3/27/12/18/39	0/29/13/18/39	0.75	
ALT (U/L) ^	69 +/− 74	72 +/− 59	0.78	
Platelet count (×10^9^/L) ^	173 +/− 71	182 +/− 63	0.47	
APRI ^	0.9 +/− 0.7	1.1 +/− 1.6	0.30	
FIB-4 ^	3.3 +/− 2.6	3.8 +/− 3.3	0.43	
HIS ^	35.8 +/− 7.1	38.7 +/− 8	0.042	
HSI > 41 (%)	18	34	0.01	
VCTE (kPa) (n = 118) ^	11.9 +/− 9.8	13.4 +/− 11.1	0.48	
VCTE > 9 (kPa) (%)	47	48	0.89	
VCTE > 11 (kPa) (%)	38	42	0.76	
CAP (dB/m) (n = 92) ^	196 +/− 37	278 +/− 36	<0.0001	
BMI (kg/m^2^) ^	26.9 +/− 6	29.8 +/− 6.5	0.013	0.01; 1.08 (1.01–0.15)
Diabetes (%)	17	26	0.24	
Hypertension (%)	43	28	0.12	
Obesity (% BMI ≥ 30 kg/m^2^)	52	26	0.004	0.004; 3.11 (1.43–6.82)
Triglyceride level (mg/dL) ^	108 +/− 97	158 +/− 97	0.01	
Metabolic syndrome (%) **	25	48	0.01	
CD4 count (cells/mm^3^) ^	582 +/− 288	756 +/− 410	0.005	
cART				
NRTI (%)	91	92	0.88	
NNRTI (%)	17	26	0.23	
PI (%)	24	26	0.78	
II (%)	73	58	0.08	

^ Mean +/− standard deviation. MLR = multilinear regression model; OR = odds ratio; CI = confidence interval; ALT = alanine aminotransferase; APRI = aspartate aminotransferase to platelet ratio index ((aspartate aminotransferase level (U/L)/aspartate aminotransferase upper limit of normal (U/L))/(platelet count (×10^9^/L) × 100); FIB-4 = fibrosis 4 index for liver fibrosis ((age (years) × aspartate aminotransferase level (U/L))/(platelet count (×10^9^/L) × square root of alanine aminotransferase level (U/L)); HSI = hepatic steatosis index (8 × (alanine aminotransferase level (U/L)/aspartate aminotransferase level (U/L)ratio) + BMI (+2, if female; +2, if diabetes mellitus)); VCTE = vibration-controlled transient elastography; kPa = kilopascal; CAP = controlled attenuation parameter; dB/m = decibel per meter; BMI = body mass index; CD4 = cluster differentiation 4; cART = combined antiretroviral therapy; NRTI = nucleoside reverse transcriptase inhibitor; NNRTI = non-nucleoside reverse transcriptase inhibitor; PI = protease inhibitor; II = integrase inhibitor. * By liver biopsy: 0 = no fibrosis; 1 = mild fibrosis; 2 = moderate fibrosis; 3 = severe fibrosis; 4 = cirrhosis. ** The presence of at least three of the following: BMI ≥ 30 kg/m^2^, diabetes, hypertension, or dyslipidemia (triglyceride level ≥ 150 mg/dL).

**Table 4:** Sensitivity analysis of the HSI (>41) versus CAP at different thresholds.

CAP Threshold dB/m (n = 92)	Sensitivity (%)	Specificity (%)	PPV (%)	NPV (%)
263	39	90	50	86
248	55	82	43	88
238	72	74	40	92

HSI = hepatic steatosis index (8 × (alanine aminotransferase level (U/L)/aspartate aminotransferase level (U/L) ratio) + BMI (+2, if female; +2, if diabetes mellitus)); CAP = controlled attenuation parameter; dB/m = decibel per meter; PPV = positive predictive value; NPV = negative predictive value.
